# Regional education on endoscopic surgery using a teleconference system with high-quality video via the internet: Saga surgical videoconferences

**DOI:** 10.1186/s12909-020-02215-0

**Published:** 2020-09-24

**Authors:** Tatsuya Manabe, Mitsuhiro Takasaki, Takao Ide, Kenji Kitahara, Seiji Sato, Seiji Yunotani, Yoshimi Hirohashi, Akihiro Iyama, Masahiko Taniguchi, Toshiro Ogata, Shuji Shimizu, Hirokazu Noshiro

**Affiliations:** 1 Department of Surgery, Faculty of Medicine, Saga University, 5-1-1 Nabeshima, Saga, 849-8501 Japan; 2 Saga University Organization for General Education, Saga University, 5-1-1 Nabeshima, Saga, 849-8501 Japan; 3 Department of Surgery, Saga Medical Center Koseikan, 400 Nakabaru, Kasemachi, Saga, 849-8571 Japan; 4 Department of Surgery, Japanese Red Cross Karatsu Hospital, 2430 Watada, Karatsu, Saga, 847-8588 Japan; 5 Department of Surgery, Takagi Hospital, 141-11 Sakemi, Okawa, Fukuoka, 831-0016 Japan; 6 Department of Surgery, Oda Hospital, 4306 Takatsuhara, Kashima, Saga, 849-1311 Japan; 7 Department of Surgery, Saint Mary Hospital, 422 Tsubukuhonmachi, Kurume, Fukuoka, 830-8543 Japan; 8 International Medical Department, Kyushu University Hospital, 3-1-1 Maidashi, Higashi-ku, Fukuoka, 812-8582 Japan

**Keywords:** Endoscopic surgery, Videoconference, Remote, High-speed internet

## Abstract

**Background:**

Effective education about endoscopic surgery (ES) is greatly needed for unskilled surgeons, especially at low-volume institutions, to maintain the safety of patients. We have tried to establish the remote educational system using videoconference system through the internet for education about ES to surgeons belonging to affiliate institutions. The aim of this manuscript was to report the potential to establish a comfortable remote educational system and to debate its advantages.

**Methods:**

We established a local remote educational conference system by combining the use of a general web conferencing system and a synchronized remote video playback system with annotation function through a high-speed internet.

**Results:**

During 2014–2019, we conducted 14 videoconferences to review and improve surgeons’ skills in performing ES at affiliated institutions. At these conferences, while an uncut video of ES that had been performed at one of the affiliated institutions was shown, the surgical procedure was discussed frankly, and expert surgeons advised improvements. The annotation system is useful for easy, prompt recognition among the audience regarding anatomical structures and procedures that are difficult to explain verbally.

**Conclusions:**

This system is of low initial cost and offers easy participation and high-quality videos. It would therefore be a useful tool for regional ES education.

## Background

Endoscopic surgery (ES)—a minimally invasive surgery introduced during the 1990s—has become popular worldwide. In Japan, the number of ESs has rapidly increased, especially since being covered by insurance. To date, ES has been undertaken at most hospitals and medical centers [1]. However, there are still differences in short-term and long-term outcomes among institutions and surgeons, depending on their experience (based on the volume of ESs undertaken) and the training environment [2–4]. Because the complicated techniques used in ES necessitate long learning curves [5, 6], an effective educational system should be devised and systematically conducted for surgeons unskilled in ES procedures, especially at low-volume institutions, to ensure patients’ safety.

Various approaches to educating new and inexperienced surgeons regarding ES have been reported, including the use of textbooks, published papers, videos, simulators, surrogate animals, and cadavers [7, 8]. Among them, high-quality videos of ES procedures, which allow surgeons to watch an operation recorded in progress, have great potential to enhance surgical education [9–12]. The conference, in which expert surgeons review ES videos and instruct the precise procedure to the audience, could not only improve the trainee’s surgical skill and knowledge, but educate the conference participants as well.

In the past, the Saga University Department of Surgery sent medical/surgical staff to affiliated institutions to educate their surgeons and staff. However, there are three problems with that approach to ES education. First, most affiliated institutions are small or medium-sized, and in some, the volume of ES is too small for training and education. Second, an expert surgeon cannot always supervise ES performed by trainees in affiliated institutions. Third, surgeons belonging to affiliated institutions are difficult to gather in one place for an educational conference because of the location of the institution. Therefore, the education for ES procedure for surgeons at affiliated institutions of Saga University was the urgent necessity.

Teleconference system has been applied to medicine for more than 30 years [13]. With the development of information technology, some authors have reported the teleconference and/or telemedicine using transmitted moving images in the early stage [14, 15]. However, it was impossible to avoid compression of images because of the limitation of transmitted information volume, resulting in decreasing the quality of moving images [16]. Therefore, the tele-education for ES could not has become popular. In 1999, Digital Video Transport System (DVTS), which is a remote conference system capable of transporting uncompressed high-quality videos without time delay or noise, was invented in Japan, and many remote conferences using DVTS has been conducted among leading institutions [17–20]. However, for a teleconference using DVTS, a special network condition, such as static IP address and 30Mbps high-speed band, needs to be established, and therefore small or medium-sized institutions are still difficult to easily join the teleconference with high-quality video,

Therefore, we planned more easily available surgical videoconferences using a teleconference system to assess the quality of ES performance and to educate the surgeons at each affiliated institution. We report the potential to establish a comfortable remote educational environment by combining the use of a general web conferencing system and a synchronized remote video playback system with annotation function that is shared via high-speed internet.

## Methods

**Fig. 1:**
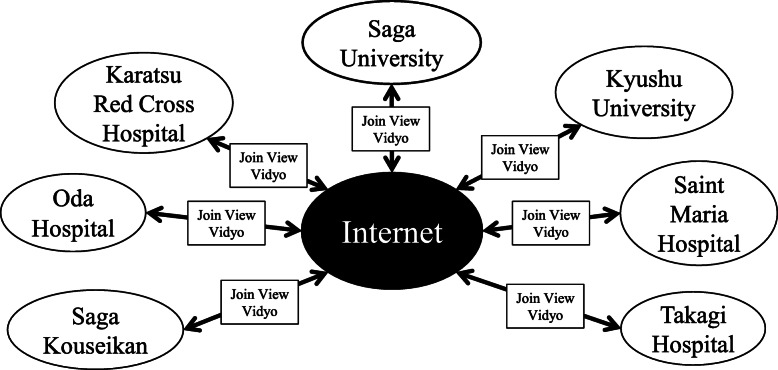
Network system of interactive teleconferences among institutions

Our remote ES educational endeavor was conducted using two systems. One is a general web conferencing system, Vidyo® (Vidyo Inc., Hackensack, NJ, USA), whose main server has been installed at Kyushu University. The other is a web-based video playback system, JoinView®, which is a system used for high-speed collaborative editing at multiple locations in the broadcasting industry. JoinView® has been expanded for medical use by Saga University, Kyushu University and the developer Unixon Systems. Each institution was connected through commercial internet [21]. The interactive teleconferences involved recorded, uncut video with bidirectional discussion via JoinView and Vidyo (Fig. 1).

**Fig. 2:**
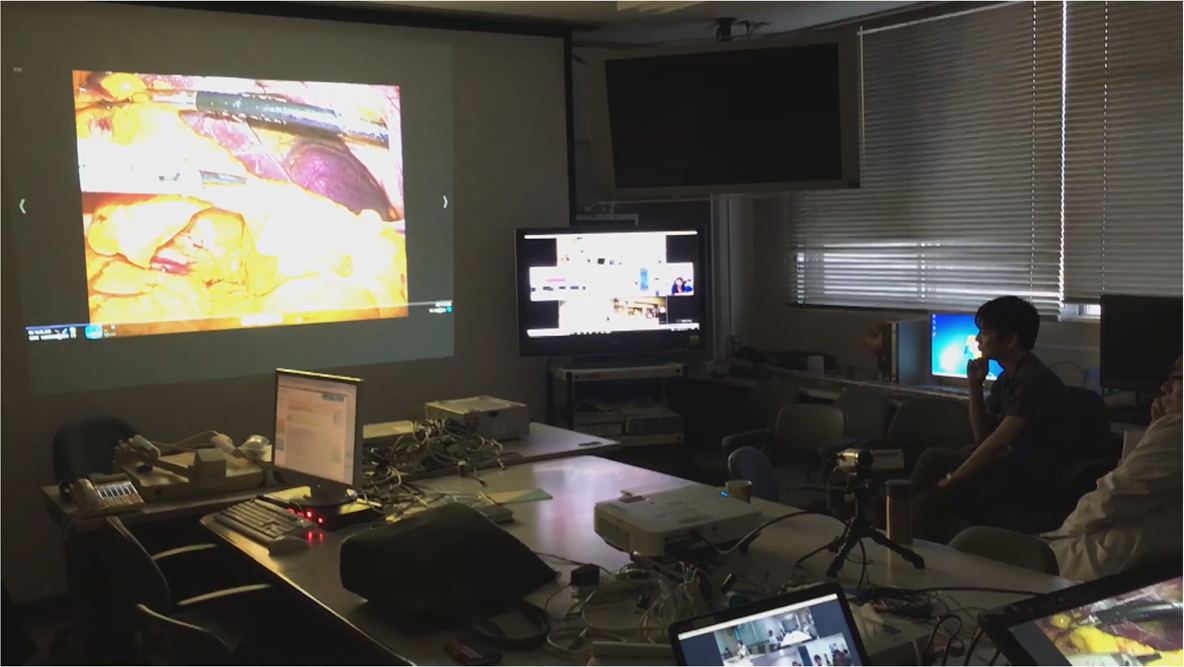
Conference room at Saga University. Left monitor: Video of ES was played with surgeon’s comment; Right monitor: View of the conference rooms at remote institutions

To prepare for a conference, the engineers first checked the transmission state, and one presenter belonging to one institution joining the conference uploaded a recorded uncut video of recently performed ES. At the conference, while showing a video, a presenter explains his procedures and proceeds with identifying the surgical anatomy. Experts then review the surgery and offer advice about the ES procedure (Fig. 2). Any participant at any institution engaged in the conference can freely ask questions about the surgery from anywhere on the network. Patients’ names are never shown on the screen to protect their privacy, and all other private information is strictly controlled.

## Results

**Fig. 3:**
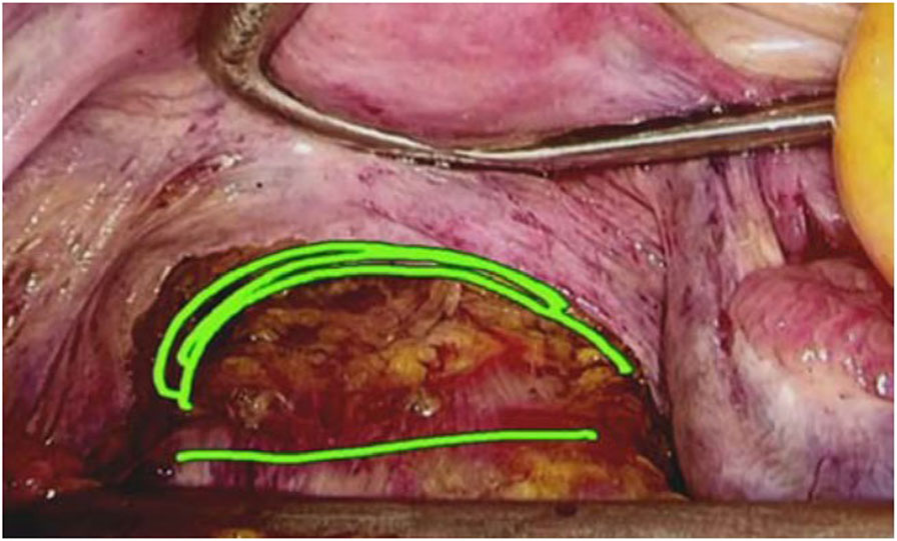
Annotation system of dots, lines, and words that explain visually on the screen what may be described verbally. Annotations can be added from anywhere by anyone on the network

Between September 2014 and September 2019, we conducted 14 videoconferences that reviewed the following laparoscopic surgical procedures: distal partial gastrectomy (*n* = 6), sigmoidectomy (*n* = 4), and low anterior resection of the rectum (*n* = 4). Details of the conferences are summarized in Table 1. For the first to sixth conferences, six institutions, including Kyushu University, were connected, and the senior surgeons belonging to one institution presented their uncut videos to assess the quality of ES at each institution. At the seventh conference, seven institutions were connected, and young surgeons volunteered to present their uncut videos for review and advice about their procedures. An annotation system, developed in JoinView, enabled dots, lines, and words to be described on the screen to clarify the verbal description. These annotations can be added from everywhere on the network (Fig. 3).

**Table 1:** Details of the 14 Saga surgical videoconferences (2014–2019)

No.	Year	Date	Presenter	Institution	Type of surgery
1	2014	9/30	Senior	Kouseikan	Distal gastrectomy
2		12/2	Senior	Karatsu	Low anterior resection
3	2015	2/24	Senior	Takagi	Distal gastrectomy
4		6/30	Senior	Oda	Distal gastrectomy
5		10/27	Senior	Saga Univ.	Intersphincteric resection
6	2016	4/26	Senior	Kouseikan	Distal gastrectomy
7	2017	1/31	Young	Karatsu	Sigmoidectomy
8		5/30	Young	Takagi	Low anterior resection
9		9/26	Young	Oda	Low anterior resection
10	2018	2/6	Senior	Saint Maria	Distal gastrectomy
11		6/5	Young	Saga Univ.	Sigmoidectomy
12		10/2	Young	Kouseikan	Sigmoidectomy
13	2019	5/28	Young	Karatsu	Distal gastrectomy
14		10/29	Young	Takagi	Sigmoidectomy

## Discussion

Currently, ES has become the mainstream choice for minimally invasive gastrointestinal surgery in Japan. Many young surgeons are eager to improve their ES skill, resulting in more qualified surgeons entering the ranks of the skilled ES qualification system [22, 23]. Various meetings for reviewing videos—so-called video clinics—have been held for a fee or free under the sponsorship of surgical societies, ES-related corporations, and personal volunteers. However, many surgeons, especially surgeons belonging to local, small institutions, cannot easily participate in such meetings because of their daily work schedules, and the long distances and expenses involved in travel. Our teleconference system could save time and travel expenses for those busy doctors [24, 25], allowing many more surgeons at various institutions to easily attend these educational meetings. Likewise, this conference system would be expected to be applied to the education for other complicated medical technique, such as microsurgery, robot-assisted surgery, endoscopic submucosal dissection, and so on.

Invention of DVTS, which could transfer uncompressed, high-quality video that is as clear as that obtained in the operating room, opened a new era for telemedicine, and many domestic and international teleconferences using DVTS have been successfully conducted among the leading institutions [17–20]. However, regional small or medium-sized institutions still could not join easily because of the special network setting for DVTS. Nowadays, several web conference systems, such as Vidyo and Zoom, have become easily available over commercial internet, but the videos brought from such systems are not suitable for surgical video conference in the absence of multiaccess handling and annotation system. Therefore, we tried to separately use a general web conference system and a synchronized remote video transfer system ensuring high-quality images. We adopted JoinView as a video transfer system and Vidyo as a teleconference system. JoinView has several advantages as follows. It can be operated with a web browser and used with various computer operating systems because no special software is required. The high-quality moving images provide clear views of the anatomy with its fine structures for the audience. Moreover, the anatomical structures and procedures are difficult to explain verbally, but by using the annotation system equipped in JoinView, the educator’s verbal descriptions are easily and immediately understood by the audience.

Uncut videos have been used during these teleconferences because edited videos, which are useful for learning the basic procedure, usually do not cover procedures in their entirety, and there is no opportunity to ask questions. When uncut video is used via telecommunication, the observers’ questions and any misunderstandings of the presenter can be addressed and clarified throughout the surgery. The ability of skilled surgeons to correct any mistakes or advice is useful not only for the presenter but for the audience as well. All expert surgeons who gave advice at our conferences were volunteers belonging to participated institutions.

As for initial investment, this system requires only common and widely available equipment, such as digital video cameras, personal computers and monitors, all usually available in most medical institutions. Moreover, the internet is economical and easy to access, and the Vidyo server at Kyushu University can be freely used for academic purposes. From the above requirement, various institutions could easily participant the teleconference with low cost.

## Conclusion

We have developed and conducted regional surgical videoconferences using a broadband internet-based telecommunication system that can preserve the original quality of surgical video. This system comes at a low initial cost, promotes easy participation, and offers high-quality video. We therefore believe that it would be a useful tool for regional education concerning ES.

## Data Availability

The author can confirm that all relevant data are presented within the manuscript.

## Abbreviations

ES: Endoscopic surgery
DVTS: Digital Video Transport System
